# Unraveling the source of corrosive microorganisms from fracturing water to flowback water in shale gas field: evidence from gene sequencing and corrosion tests

**DOI:** 10.3389/fmicb.2025.1552006

**Published:** 2025-06-18

**Authors:** Yanran Wang, Shaomu Wen, Shibo Zhang, Yongfan Tang, Xi Yuan, Fang Guan, Jizhou Duan

**Affiliations:** ^1^Research Institute of Natural Gas Technology, Petrochina Southwest Oil & Gasfield Company, Chengdu, China; ^2^Petrochina Southwest Oil & Gasfield Company, Chengdu, China; ^3^Sichuan Huayou Group Corporation Limited, Petrochina Southwest Oil & Gasfield Company, Chengdu, China; ^4^State Key Laboratory of Advanced Marine Materials, Chinese Academy of Sciences, Qingdao, China; ^5^Guangxi Key Laboratory of Marine Environmental Science, Institute of Marine Corrosion Protection, Guangxi Academy of Sciences, Nanning, China

**Keywords:** microbially influenced corrosion, fracturing water, flowback water, weight loss, shale gas resources

## Abstract

As an insidious and often underestimated phenomenon, microbially influenced corrosion (MIC) poses a significant threat to the integrity and longevity of oil and gas pipelines. However, the complex corrosive microorganisms, that might induce MIC in underground pipelines, might be introduced by the fracturing water during the production period, or they may also exist in the native corrosive microbial community underground. In this study, microbial community analysis was conducted to unravel the source of corrosive microbes in oil and gas pipelines. Meanwhile, the corrosion rate caused by the fracturing water and the flowback water on steel were studied via combining electrochemical analysis and weight loss analysis. Three types of fracturing fluids and the flowback water were analyzed based on 16S rRNA gene sequencing. Bacteria with multiple metabolic functions, including sulfate-reducing bacteria, acid producing bacteria, petroleum oil-degrading bacteria, and nitrate-reducing bacteria, were found in the flowback water. Comparative analysis on the fracturing fluids and the flowback water showed that corrosive *Thermodesulfobacterium* and *DesulfobacterSota* originated from the underground rocks. While other microorganisms such as *Desulfomicrobium*, *Acinetobacter* and *Acetobacterium* may be introduced via the fracturing water. The weight loss of steel coupons in fracturing and flowback water were 35.04±7.57 mpy and 28.07±4.49 mpy, respectively. The corrosion weight caused by the fracturing water may accounts for 75.16% of the whole corrosion during the 5 days’ immersion under laboratory conditions. The results provide a reference for tracing the sources of corrosive microorganisms and controlling microbially induced corrosion in shale gas resources.

## Graphical abstract

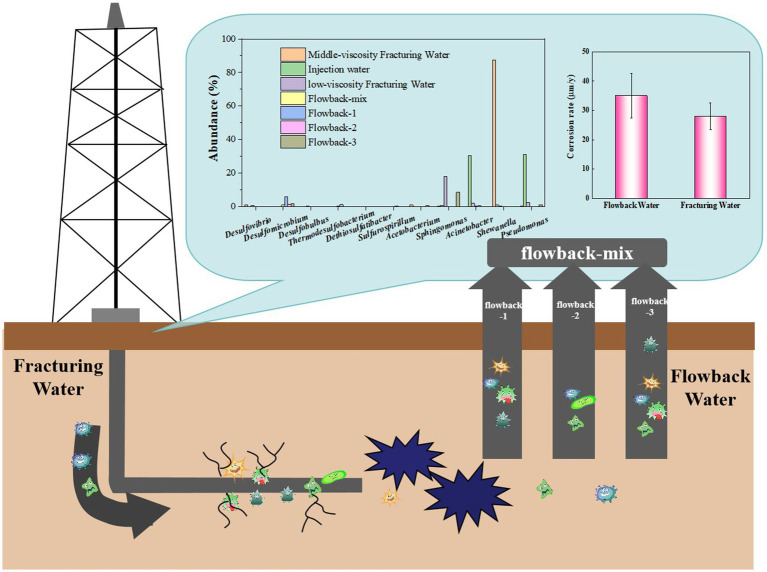

## 1 Introduction

Shale gas resources are an important energy sources for industrial and economic development. However, the conditions for shale gas extraction are incredibly intricate, leading to particularly intractable issues with the corrosion of infrastructure and machinery. The economic impact of corrosion accounts for about 3.34% of China’s GDP (Hou et al., 2017). The incidence of accidents due to corrosion within the critical submarine pipeline transportation systems can be as high as 37% (Honghong and Guoheng, 2017). For the long-term service of shale gas field pipelines, the occurrence of pipeline failure due to corrosion can lead to substantial economic losses and pose significant safety hazards. However, with the development of shale gas, some gas gathering station pipelines have successively exhibited perforation phenomena due to corrosion. For example, the Alaskan oil pipeline leak in the United States was contributed to be caused by microbially influenced corrosion (MIC) (Jacobson, 2007). At least 20% of accidents in the oil and gas industry are due to internal corrosion caused by subsediment corrosion (UDC) (Sliem et al., 2021). Such sediments often contain complex microbial communities mixed with oil, water and sediment deposits (Wang Z. et al., 2020).

The environments in shale gas field are usually complex and harsh, characterized not only by high temperatures, pressures, salinity, and mineralization but also by a rich diversity of microbial symbiosis (Hull et al., 2018). In those harsh and dynamic environments, pipelines are subjected to a variety of corrosive factors. Among these, MIC is particularly challenging due to its undetectable and progressive nature (Xu et al., 2023; Flemming et al., 2024). Microorganisms such as sulfate-reducing bacteria (SRB) (Guan et al., 2021), acid-producing bacteria (APB) (Zhang et al., 2025), nitrate reducing bacteria (NRB), iron-reducing bacteria (IRB) (Okamoto et al., 2012), iron-oxidizing bacteria (IOB) and oil degrading microorganisms thrive in the nutrient-rich underground conditions were often found within underground pipelines, exacerbating the corrosion process. With the continuous interaction between various corrosive microbes and pipelines, there is often a cycle of elements such as iron, nitrogen, carbon, and sulfur. Microorganisms tend to adhere onto the surfaces of the metals and induce local corrosion on metals surfaces. Those microorganisms produce extracellular polymeric substances (EPS), hydrogen sulfide, acetic acid, carbon dioxide and other metabolic by-products. These by-products attacked metal surfaces via chemical reactions, accelerating the direct corrosion of metals. Besides, some electroactive microorganisms (EAMs) (Deng et al., 2015; McCully and Spormann, 2020) might directly extract electrons from the metals directly, and induce the accelerated corrosion.

Corrosive microorganisms in underground shale gas pipelines emanate from two principal sources (Lenhart et al., 2014; Hou et al., 2022). On the one hand, they are derived from the deep subsurface petroleum reservoirs. On the other hand, they may be introduced exogenously during the shale gas field extraction phase by hydraulic fracturing water. Hydraulic fracturing, a commonly employed technique for the development and enhancement of shale gas production, is extensively utilized for the modification and production of the shale gas production (King, 2012). The performance of fracturing water plays an exceedingly critical role in the post-fracturing production enhancement effects (Wang et al., 2023; Gao et al., 2024). On the one hand, the fracturing water is capable of transporting proppant and filling it within the fractures, thereby creating a high-conductivity fracture that exceeds the reservoir’s permeability. On the other hand, the fracturing water possesses a filtrate loss characteristic; it can seep through the fracture walls into the reservoir’s matrix, potentially inducing phenomena such as clay swelling, pore plugging, and alterations in permeability. Water-based fracturing water has water as its main component, and it can increase viscosity by forming physical association or chemical crosslinking between water-soluble molecules, so as to better meet the requirements of sand carrying (Xue et al., 2021; Wan et al., 2023). In the use of fracking water, a large amount of water, chemicals, and proppants (such as, sands) are needed to be mixed to fracture and maintain open fractures in low-permeability shale and tight formations (Orangi et al., 2011). The interaction between the fracking water and the shale will create a region characterized by strong capillarity and permeability changes near the fracture, in order to extract shale gas resources from the rock fissures underground. Among them, the main components of polymer fracking water include polymer thickener, crosslinking agent, temperature stabilizer, and breaker.

In the flowback water, due to reasons such as dissolving stratum substances during the staying period, it often contains a high concentration of total dissolved solids, heavy metals, organic matter, etc. (Jackson et al., 2013; Hull et al., 2018). Some may even contain radioactive substances. In these flowback waters, separation and purification technologies are used to separate gas from sandstone and further purify them (Dickhout et al., 2017). In addition, some rock cuttings and fracturing water might also be present in these flowback waters in addition to the underground gas (Dickhout et al., 2017; Wang B. et al., 2020).

Meanwhile, some corrosive bacteria are thought to enter underground fields via fracking water. Contaminated water sources in pipeline systems can introduce corrosive microbes into subsurface pipelines, causing corrosion. In shale gas underground environments, abundant organic carbon sources fuel microbial growth, accelerating corrosion. The underground microbial community is diverse. SRB use hydrogen, organic acids, etc. as electron donors to reduce sulfate ions, generating corrosive sulfides and organic acids (Muyzer and Stams, 2008). Sulfur oxidizing bacteria (SOB) (Gao and Fan, 2023) can oxidize sulfur-containing compounds, affecting the pipeline’s corrosion environment. Besides SRB and SOB, saprophytic bacteria break down organic matter (Huber et al., 2014; Gao and Fan, 2023). IOB (Liu et al., 2025) could oxidize iron, which results in the formation of iron oxides and hydroxides on pipelines and thereby accelerates corrosion. These microorganisms have symbiotic relationships and affect pipeline corrosion through metabolic pathways (Mand and Enning, 2021; Wang et al., 2023). SRB and SOB interact in a sulfur-cycle symbiosis; SRB produce hydrogen sulfide, and SOB oxidize it, altering the corrosion rate. Saprophytic bacteria provide nutrients, enhancing the corrosion-promoting effect. IOB-generated iron oxides act as electron acceptors for SRB, accelerating corrosion. Fungi form biofilms with bacteria, and their extracellular substances influence bacterial adhesion and growth, thus affecting corrosion.

The characteristics of the bacterial communities in shale gas fields are crucial for monitoring and controlling microbial activities within these environments (Zhou et al., 2020; Tang et al., 2024). Through microbial analysis, the subsurface corrosive microbial conditions of shale gas fields can be assessed, and corresponding measures can be taken to optimize the extraction process and environmental protection (Zhang Q. et al., 2023). With the advancement of microbial and molecular microbial technologies, research into the bacterial communities of shale gas fields is continuously deepening.

In this present study, the microbial community of the fracturing water and flowback water from a certain site was analyzed via 16S rRNA gene sequencing technology, aiming to trace the origination of corrosive microbes in shale gas pipelines. Besides, electrochemical measurements and weight loss tests were also performed to compare the corrosion rate caused by fracturing and flowback water, respectively. This study contributes to find the origination of corrosive microbes and determine the extent of corrosion losses caused by the fracturing water, providing a scientific basis for the sustainable development of shale gas fields.

## 2 Experiments and methods

### 2.1 Sample collection

Seven fracturing water and flowback water samples were collected from a certain site of the Sichuan shale gas fields, China. Based on the actual operating condition, the whole fracturing water is a mixed solution composed of bulk water, middle-viscosity fracturing water and low-viscosity fracturing water. The bulk water, labeled as injection water, is used to diluting the fracturing water. Thus, all the fracturing water group including the middle-viscosity fracturing water and low-viscosity fracturing water and the injection water were sampled. Based on the actual operating condition, the flowback water is a mix of several underground pipelines, named as flowback-1, flowback-2 and flowback-3, separately. Excepts for flowback-1, flowback-2 and flowback-3 pipelines, the flowback water of flowback-mix comprises other flowback waters from other pipelines. The mixed flowback water is labeled as flowback-mix. All the flowback water were also sampled. The detailed information of the fluid samples were shown in Supplementary Table S1. The liquid samples were stored in sterile polytetrafluoroethylene (PTFE) bottles, sealed, and kept at low temperature. They were then transported to the laboratory for analysis as soon as possible at 4°C. Samples were filtered through 0.22 μm acetate fiber filter membrane to obtain microbial cells before DNA extraction.

### 2.2 DNA extraction and quality checked

All filter membrane samples were cut into pieces in a sterile laminar flow hood and placed into polycarbonate (PC) tubes. Subsequently, the cells were lysed using magnetic beads under a condition of 6 m·s^−1^ for 40 s. Microbial genomic DNA was extracted using E.Z.N.A. Soil DNA Kit (Omega Bio-tek, Inc., USA) following the manual. Concentration and quality of the genomic DNA of samples were verified as qualified by NanoDrop 2000 spectrophotometer (Thermo Scientific Inc., USA). The concentration of the sample DNA was higher than 10 ng·μL^−1^, and the A_260_/A_280_ ratio was around 1.80. DNA samples were stored at −20°C for subsequent experiments.

### 2.3 PCR amplification

The V3-4 hypervariable region of bacterial 16S rRNA gene were amplified with the universal primer 338F (5′-ACTCCTACGGGAGGCAGCAG-3′) and 806R (5′-GGACTACNNGGGTATCTAAT-3′). The PCR was carried out on a Mastercycler Gradient (Eppendorf, Germany) using a 25 μL reaction volume, containing 12.5 μL 2 × Taq PCR MasterMix (Vazyme Biotech Co., Ltd., China), 3 μL BSA (2 ng·μL^−1^), 1 μL Forward Primer (5 μM), 1 μL Relative Reverse Primer (5 μM), 2 μL template DNA, and 5.5 μL ddH2O. Cycling parameters were 95°C for 5 min, followed by 28 cycles of 95°C for 45 s, 55°C for 50 s and 72°C for 45 s with a final extension at 72°C for 10 min. The PCR products were purified using a Agencourt AMPure XP Kit (Beckman Coulter, Inc., USA).

### 2.4 High throughput sequencing

Deep sequencing of the amplified fragments of the bacterial 16S rRNA gene was performed on the Illumina MiSeq/NovaSeq platform (Illumina, Inc., USA) at Beijing Allwegene Technology Co., Ltd.

### 2.5 Bioinformatics

Pear (Zhang et al., 2013) (v0.9.6) software was used to filter and splice raw data. The sequences were removed from consideration if they were shorter than 120 bp, had a low quality score (≤20), or contained ambiguous bases. During splicing, the minimum overlap setting was 10 bp, and the mismatch rate was 0.1. After splicing, Vsearch (Rognes et al., 2016) (v2.7.1) software was used to remove sequences with length less than 230 bp and the chimeric sequence was used by UCHIME method according to the Gold Database. Qualified sequences were clustered into operational taxonomic units (OTUs) at a similarity threshold of 97% using the Uparse (Edgar, 2013) algorithm of Vsearch (v2.7.1) software. The BLAST (Ye et al., 2006) tool was used to classify all OTU representative sequences into different taxonomic groups against Silva138 Database (Pruesse et al., 2007) and e-value threshold was set to 1e−5.

### 2.6 Electrochemical tests

In the oil and gas production field, the fracturing water is a mixed solution composed of injection water, middle-viscosity fracturing water and low-viscosity fracturing water. Thus, in the electrochemical and corrosion weight loss experiments, a mixture solution containing injection water, middle-viscosity fracturing water and low-viscosity fracturing water was used as named as the fracturing water in this part. Similar, the mixed flowback water (Flowback-Mix) was used in this part. To find out the corrosion behavior differences of steel induced by fracturing water and flowback water, respectively. The corrosion electrochemical tests and weigh loss experiments were taken out to find the corrosion behavior differences of coupons in fracturing water and flowback water, respectively. The electrochemical tests were conducted in a traditional three electrode electrochemical cell (Guan et al., 2024), with Q355 steel coupons serving as working electrodes, and a platinum plate and a saturated calomel electrode (SCE) as the counter and reference electrode, respectively. The Q355 steel coupons measuring 10 mm × 10 mm × 10 mm were welded to the copper wire and then sealed with epoxy resin, leaving an exposed area of 1 cm^2^. Postgate C (PGC) medium inoculated with fracturing water or flowback water were used as the media for the electrochemical measurements and weight loss tests. The composition of PGC media was the same as described in previous studies (Yu et al., 2013). Before experiment, the PGC culture media was deoxygenated by purging with high-purity nitrogen gas (with a purity of 99%) for 30 min and autoclaved at 121°C for sterilization. The electrodes were also sterilized via UV light. Each vial was inoculated anaerobically with 5% (v/v) fracturing water or flowback water and then cultured at 30°C for 5 days (Guan et al., 2024).

Electrochemical impedance spectroscopy (EIS) and linear polarization resistance (LPR) tests were performed after the open circuit potential (OCP) stabilized for 5 days using a 10 mV sinusoidal signal with frequencies ranging from 100 kHz to 10 mHz. The data were analyzed using Princeton ZSimpWin version 3.21. LPR measurements were performed at a potential scan rate of 0.125 mV/s with a range of −10 to 10 mV vs. OCP.

### 2.7 Weight loss measurements

All polished coupons were ultrasonically cleaned in anhydrous ethanol, nitrogen drying, and then weighed with an analytical balance before experiment. After the cultivation for 5 days at 30°C in PGC medium inoculated anaerobically with 5% (v/v) fracturing water or f, the steel coupons were taken out and the corrosion products were removed. The corrosion rates of the coupons were calculated according to the weights of the specimens measured before and after the experiment (ASTMG1-03, 2003).

## 3 Results and discussion

### 3.1 The bacterial community distribution of the viscosity fracturing water

The goods coverage of fracturing water is 100% all samples (Supplementary Table S2), illustrating the V3–V4 Illumina sequencing for sequence capture the core of the fracturing water microbial communities. The calculated Chao1, which represented species richness of each sample, was quite different in these samples. Chao1 index in water distribution, Low-viscosity fracturing water and Middle-viscous water were 988, 1,344, and 260, respectively.

**Figure 1:**
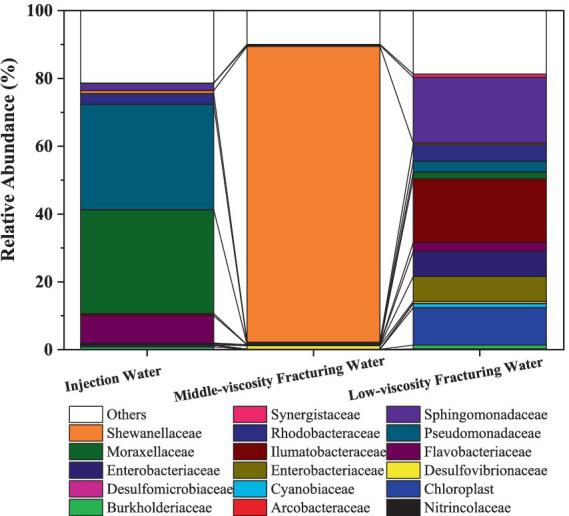
Relative abundance of 16S rRNA gene sequences of the samples at the bacterial family level.

At the phylum level (Supplementary Figure S1), Proteobacteria were the dominant microorganisms in the three samples, accounting for 74.37, 50.63 and 88.84% in the injection water, low-viscosity fracturing water and middle-viscous, respectively. *Desulfobacterota* was found in accounting for 0.18, 0.97 and 1.24% in the injection water, low-viscosity fracturing water and middle-viscous, respectively. Figure 1 shows the relative abundance of 16S rRNA gene sequences of the fracturing water samples at the bacterial family level. Microbial community abundance differed among the three solutions in the level of family, the *Moraxellaceae* (30.68%) and *Pseudomonadaceae* (31.08%) dominated in the injection water samples. While, they take only 0.12 and 0.40%, respectively, in middle-viscosity fracturing water and only 1.96 and 3.25%, respectively, in low-viscosity fracturing water. The *Shewanellaceae* occupied 87.26% in the middle-viscosity fracturing water, but only 0.45% in the low-viscosity fracturing water. *Chloroplast*, *Ilumatobacteraceae* and *Sphingomonadaceae* in the top three abundant family accounting for 11.08, 18.82, and 19.37%, respectively, in the low-viscosity fracturing water. The high abundance of *Chloroplast* in low-viscosity fracturing water might come from the other components of aqueous solution. It should be noted that corrosive SRB strains of *Desulfomicrobiaceae* and *Desulfovibrionaceae* were found in all these three solutions and occupied 0.01, 0.10%; 0.02, 1.02 and 0.04%, 0.56% in aqueous solution, middle-viscosity fracturing water and middle-viscosity fracturing water, respectively.

As an ubiquitous mixotrophic bacteria, *Arcobacteraceae* are playing important roles in carbon, nitrogen, and sulfur cycling in global oceans (Li et al., 2024), and their abundance increased in Low-viscosity fracturing water (0.23%) compared that in injection water (0.03%).

**Figure 2:**
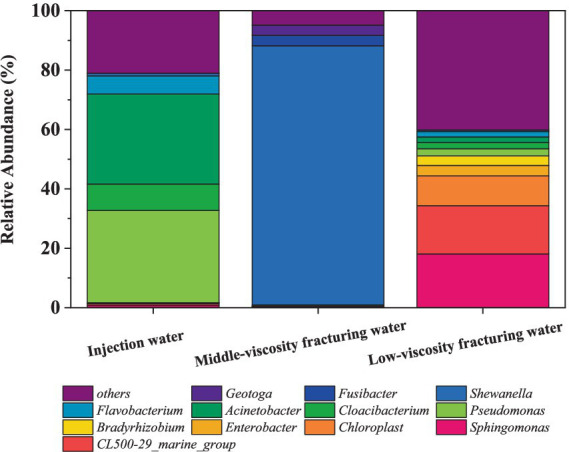
Relative abundance of 16S rRNA gene sequences of the samples at the genus level.

Figure 2 shows the abundance of microbes at genus level in fracturing water. There are significant differences in the microbial communities among different samples. At the genus level, *Pseudomonas*, which is the most common in the injection water, while in the middle-viscosity fracturing water, the most dominant genus is *Shewanella*. Among them, relevant studies have found that many *Pseudomonas* can accelerate the corrosion of iron (Li et al., 2023), and in the presence of *Pseudomonas*, the efficiency of cathodic protection will decrease (Zhou et al., 2024).

For the injection water (Figure 2), *Pseudomonas* and *Acinetobacter* dominated in the water used for hydraulic fracturing with the abundance at 31.04 and 30.07%, respectively. *Pseudomonas* is a strictly aerobic, gram-negative bacterium. It is a common oil degrading bacterium and has a good degradation effect on light crude oil (Varjani and Upasani, 2016). Its degradation efficiency for aromatic hydrocarbons is higher. *Acinetobacter* comes in second with a content of 30.37%, which is also a common oil degrading bacterium and has a good degradation effect on light crude oil (Qu et al., 2023). Additionally, chloroplasts were also discovered, with a content of 0.51%.

In the middle-viscosity fracturing water (Figure 2), the predominant microorganism in the genus level is *Shewanella*, representing 87.26% of the microbial population. As a facultative anaerobe widely found in various environments, *Shewanella* is capable of utilizing a range of electron acceptors under anaerobic conditions to carry out energy metabolism and electron transfer (Ikeda et al., 2021; Hernández-Santana et al., 2022). These include ferric iron, nitrate, nitrite, and certain heavy metal ions (Zang et al., 2023). Besides, *Sphaerochaeta*, accounting for 1.21% of the whole population. *Sphaerochaeta* is known to consume the byproducts of petroleum biodegradation by other microorganisms, participate in biofilm formation, and supply H_2_ to methanogens and other members of the community (Bidzhieva et al., 2018). *Pseudomonas* accounts for 0.4% of the microbial composition, and *Acinetobacter* represents 0.12% of the whole population. It was reported that *Pseudomonas* (Varjani and Upasani, 2016) and *Acinetobacter* (Qu et al., 2023) exhibit effective degradation on crude oil. Besides, SRB strain *Desulfovibrio* is also found at a concentration of 1.02%.

In the low-viscosity fracturing water sample (Figure 2), *Sphingomonasa*, *Acinetobacter* and *Pseudomonas* account for 18.09, 3.27, and 2.46%, respectively. Besides, *Enterobacter* is also detected at 3.56%. besides, 11.08% of the sample is attributed to *Chloroplast*, which may originate from algae or photosynthetic microorganisms potentially present in this water. However, *Shewanella* and *Desulfovibrio* were found at a relatively low abundance of 0.45 and 0.55%, respectively.

As a facultative anaerobe widely found in various environments, *Shewanella* is capable of utilizing a range of electron acceptors under anaerobic conditions to carry out energy metabolism and electron transfer (Ikeda et al., 2021). It can carry out bidirectional electron transfer between *Shewanella* cells and metals through the extracellular electron transfer (EET) process. During this process, *Shewanella* obtains electrons from metals for its own metabolic activities, thus accelerating the oxidation process of metals and leading to corrosion. In addition, *Shewanella* can also perform bidirectional electron transfer with polarized electrodes (Yang et al., 2020). Such electron transfer mainly relies on the outer membrane cytochromes and redox mediators in *Shewanella*. Studies indicated that the EET relevant genes and proteins in the Mtr pathway play a crucial role in the extracellular electron transfer process (Rowe et al., 2021). They are able to transfer electrons from inside the cells to the metal surface outside the cells.

Actually, the metabolic activities of microorganisms all the electrochemical reactions are controlled by the redox potential (Ratledge, 1994). In the complex environment of underground pipelines in oil and gas fields with high pressure and the co-existence of various microbes, there may be a variety of redox mediators. This provides various metabolic pathways and interaction potentials among different species of microorganisms. The injection of these microorganisms via fracturing water has also brought disturbances to the original microbial community into the downhole and introduced a series of possible new metabolic possibilities. In underground environments rich in salts and high temperature, it is possible that some microbes might degrade large-molecule crude oil to produce small-molecule organic substances, and these small molecules are then utilized by bacteria such as *Marinobacterium*, *Fusibacter*, and *Desulfomicrobium*, for growth and reproduction. During this process, two main processes occur simultaneously. First, energy is generated through the electron transport chain. Second, substances such as organic acids (e.g., acetic acid, lactic acid, etc.) and biological sulfides (e.g., hydrogen sulfide) are secreted. During this process, while generating energy through the electron transport chain, it will secrete substances such as organic acids (such as acetic acid, lactic acid, etc.) and biological sulfides (such as hydrogen sulfide). These organic acids can lower the pH value of the surrounding environment, creating an acidic microenvironment, thus accelerating the corrosion process of metallic materials. Especially for common industrial metallic materials like carbon steel and stainless steel, they will damage the passive film on the surface of the metal, prompting the metal to undergo an electrochemical reaction, leading to the occurrence of local corrosion phenomena such as pitting corrosion and crevice corrosion.

### 3.2 The bacterial community distribution of the flowback water

We obtained 751,819 high-quality bacterial sequences (ranging from 79,713 to 87,071). The bacterial coverage of all samples were 100%, indicating that the V3–V4 Illumina sequencing captured the core microbial communities of the samples. The composite sample from flowback-mix revealed 130 species with a Chao1 index of 151.23; the sample from flowback-1 discovered 155 species with a Chao1 index of 167.49; the sample from flowback-2 found 140 species with a Chao1 index of 170.67; the sample from flowback-2 detected 138 species with a Chao1 index of 149.48; while the sample from flowback-3 discovered 50 species with a Chao1 index of 61.25.

**Figure 3:**
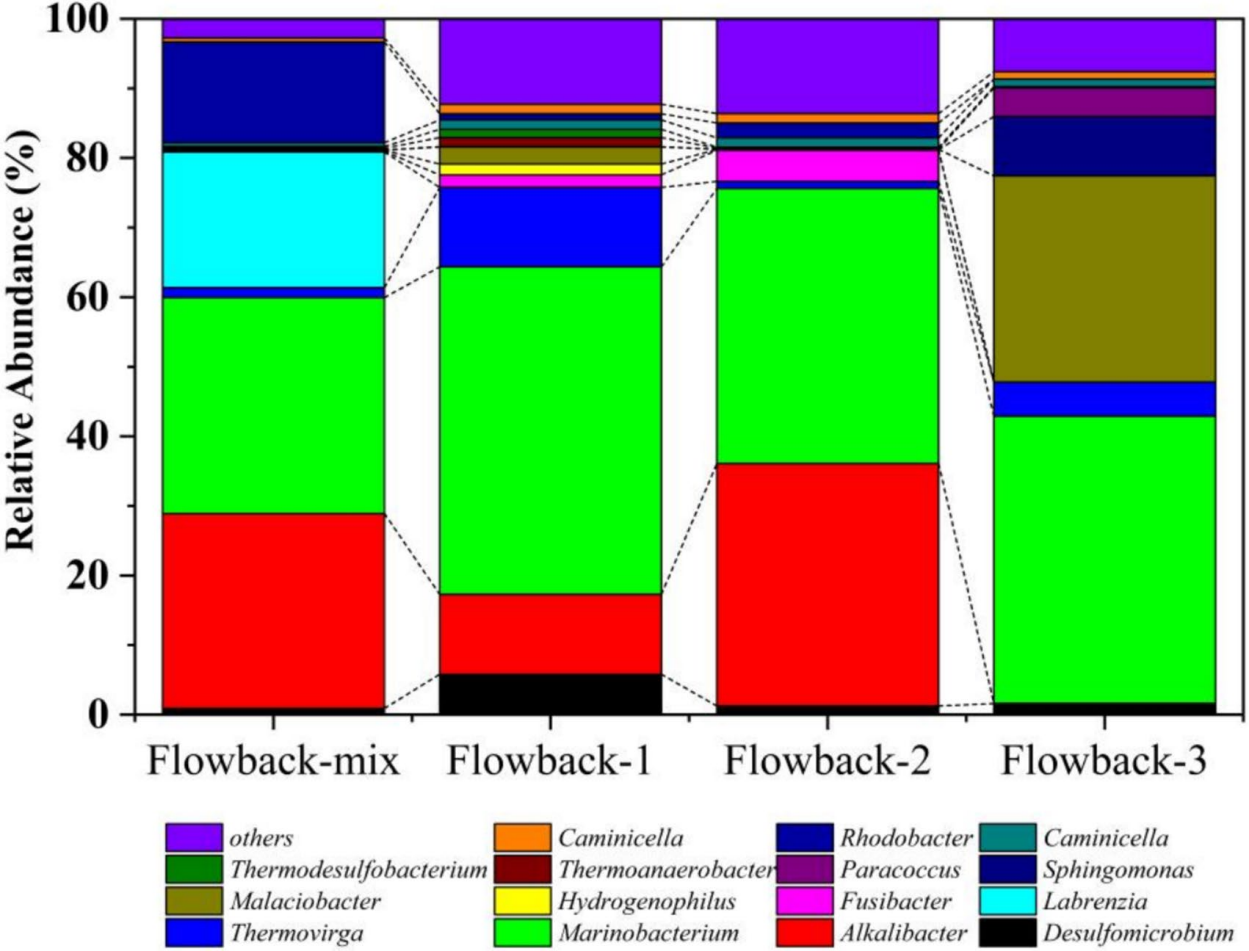
The abundance of microbial community of flowback water at the genus level.

At the genus level (Figure 3), *Marinobacterium* was the dominant bacterium in all of the flowback water, occupying 31.18, 47.72, 40.09, and 41.74% in flowback-mix, flowback-1, flowback-2, and flowback-3, respectively. The abundances of *Alkalibacter* in flowback-Mix, flowback-1 and flowback-2 are 28.16, 11.64, and 35.32%, respectively. But the abundance of *Alkalibacter* in flowback-3 is 0.01%. The flowback water of flowback-mix comprises flowback water from other pipelines excepts for flowback-1, flowback-2, and flowback-3 pipelines. *Labrenzia* has an abundance of 19.59% in flowback-mix, but almost zero in flowback-1, flowback-2, and flowback-3. Similarly, the abundance of *Rhodobacter* in 14.53% in flowback-mix. But, the abundances of *Rhodobacter* are very low in flowback-1, flowback-2, and flowback-3. It is worth noting that bacteria that are relatively heat-resistant microbes have also been found in the flowback water, such as *Thermoanaerobacter* (Weghoff Marie and Müller, 2016), *Thermodesulfobacterium* (Hamilton-Brehm et al., 2013), and *Caminicella* (Alain et al., 2002). Their presence is related to the high-temperature and high-pressure environment in the oilfield downhole (Kaster et al., 2009).

### 3.3 The electrochemical measurements

#### 3.3.1 Evolution of open circuit potential (OCP) and linear polarization resistance (LPR)

**Figure 4:**
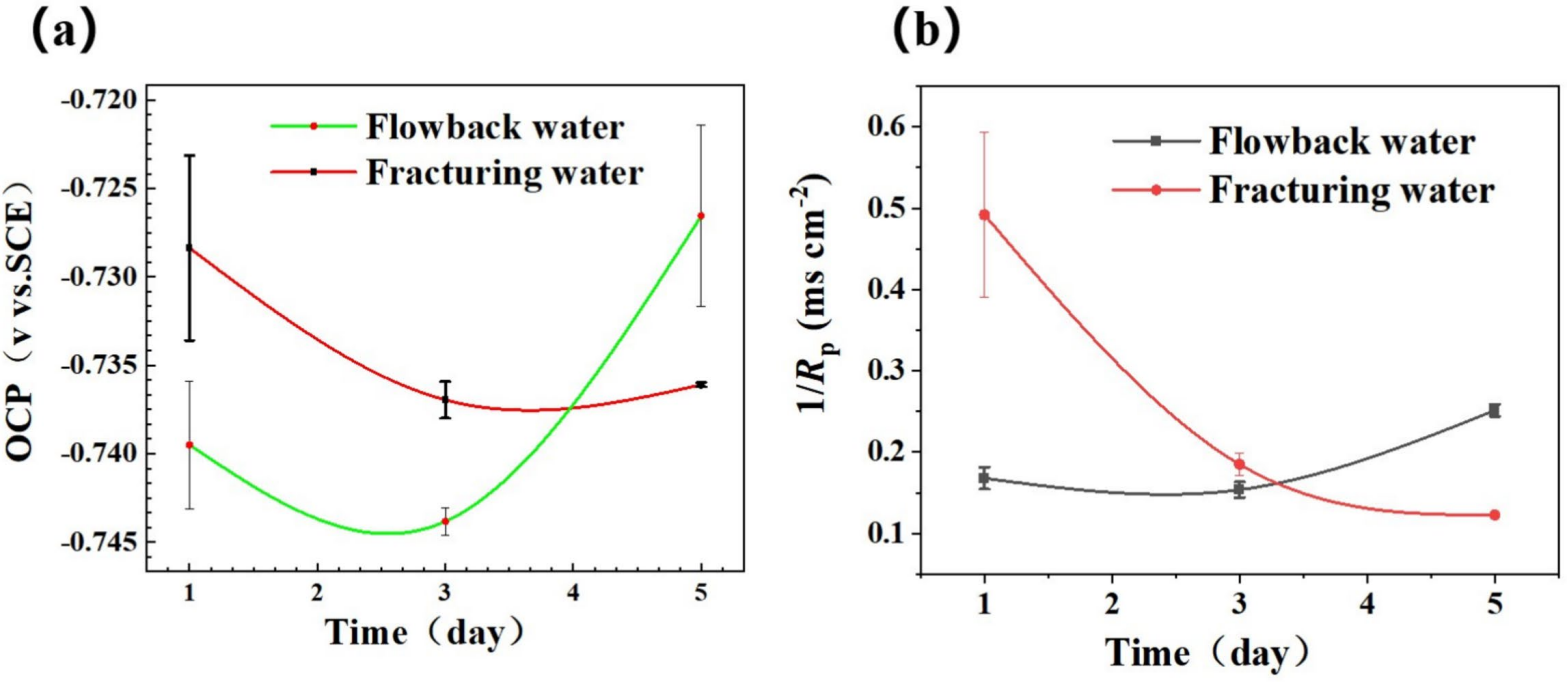
Variation of **(a)** OCP and **(b)** 1/*R*_p_ obtained by LPR measurement of steel in PGC media inoculated with fracturing water and flowback water, separately.

The variation in OCP and 1/*R*_p_ of steel coupons in different media was shown in Figure 4. Figure 4a shows the variation of OCP of the Q355 coupons in a culture medium inoculated with fracturing water and flowback water samples as bacterial sources over a period of 5 days. The OCP of the coupons in those two types of waters exhibited a trend of initial decline followed by an increase over time. Once immersed in the media, the corrosive chemicals would attack steel chemically, leading to gradual activation and subsequent corrosion. Simultaneously, the corrosive microorganisms existing in the culture medium facilitate the corrosion of the test piece by means of direct contact and the generation of metabolites. The corroded area of the test piece expands and the OCP shifts negatively in the first 3 days’ immersion. Over the following days, as the soaking time increases, the OCP of the test pieces within both systems exhibits a positive shift. This positive shift of OCP occurs when large organic molecules adhere to the surface. It is worthy of note that the magnitude of the positive shift in OCP within the fracturing water samples is considerably less than that in the flowback water samples (Guan et al., 2024). This variation in OCP might be caused by the more significant adsorption of microbes and metabolic activities.

Figure 4b shows the variation of the reciprocal polarization resistance (1/*R*_p_) values, representing the instantaneous corrosion rate of Q355 steels in different media. For the coupons in culture media inoculated with fracturing water, the values of 1/*R*_p_ was relatively high on the 1st day’ immersion. However, it decreased sharply after 3 days, suggesting that the corrosion rate was gradually decreased. There may be certain protective effects in the backflow water due to microorganisms or some chemical components, and the protective effect weakened as time went by. While for the coupons immersed in culture media inoculated with flowback water, the values of 1/Rp stayed at a relatively low level during the first 3 days. However, it increased on the 5th day, indicating that the corrosion rate was low during this period. From the third day to the fifth day, it gradually decreased, suggesting that the corrosion rate was gradually increasing.

#### 3.3.2 Electrochemical impedance spectroscopy (EIS)

**Figure 5:**
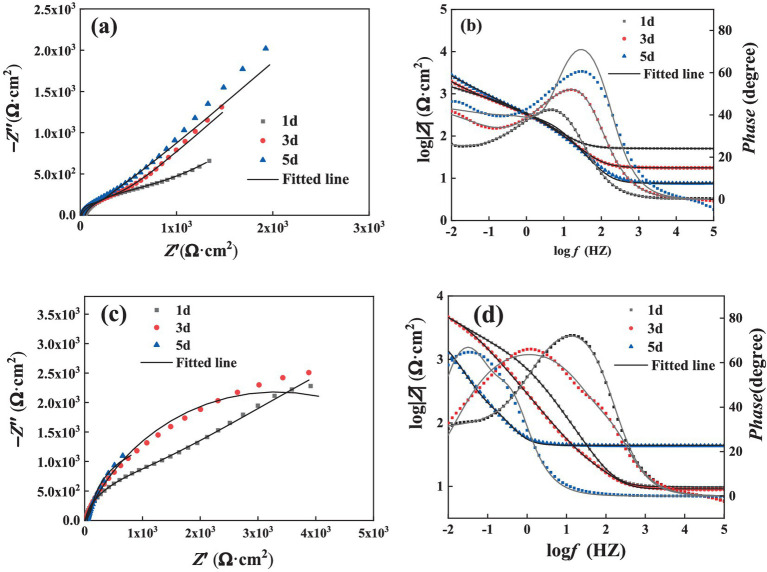
EIS spectra of steel in the presence of **(a,b)** fracturing water or **(c,d)** flowback water: **(a,c)** Nyquist plots and **(b,d)** Bode plots.

**Figure 6:**
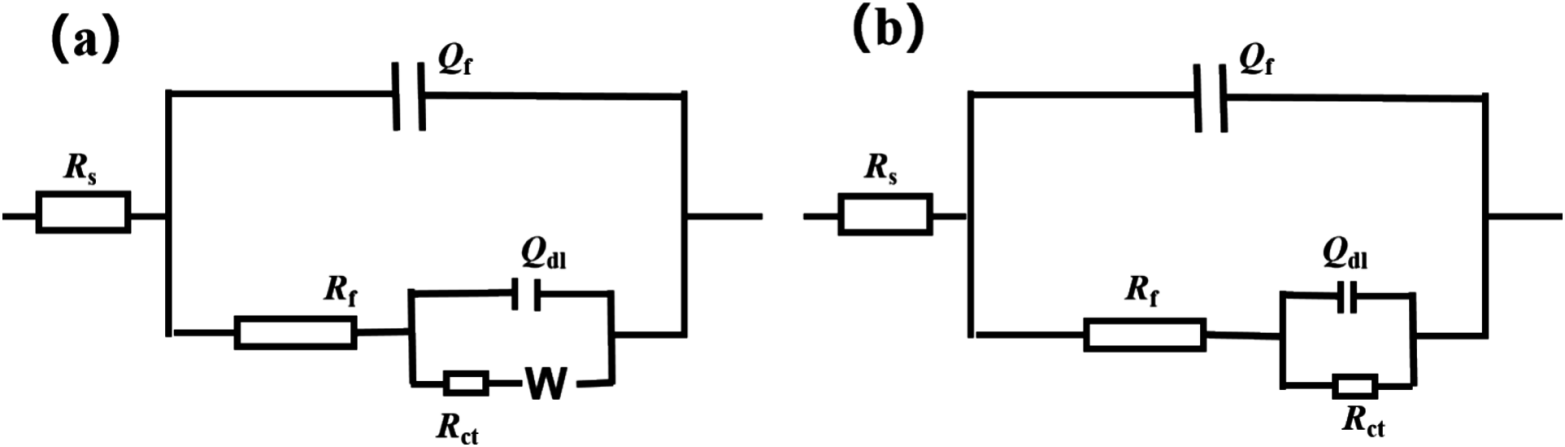
Fitting circuit employed for the EIS results of the coupons in fracturing water **(a)** and flowback water **(b)**. *R*_s_: the resistance of the electrolyte solution; *Q*_dl_: the constant phase element of electrical double layer; *R*_ct_: the charge transfer resistance of electrical double layer; *Q*_f_: the constant phase element of mixture of biofilm and corrosion product; *R*_f_: the charge transfer resistance of mixture of biofilm and corrosion product. *W*: the Warburg impedance.

Figure 5 illustrates the Nyquist plot and Bode plot of the Q355 coupons immersed in a culture medium inoculated with fracturing water samples and flowback water samples, respectively. All the electrochemical data are fitted using model shown in Figure 6, and the corresponding electrochemical fitting data are presented in Table 1 (fracturing water) and Table 2 (flowback water). The *R*_ct_ of the test coupons within each system exhibited a decreasing trend over time, whereas the *R*_s_ and the *R*_f_ remained relatively stable during the immersion period. The *Q*_f_ demonstrated an increasing trend over time, which might be induced by the progressively formation of the microbial corrosion film and corrosion product film of the test coupons in both systems. The corrosion product film harbors conductive corrosion products such as FeS, thereby may induce the fluctuation of *R*_ct_ of the test coupons. Bode plot analysis revealed that with an increase in the immersion time of the test coupons, the phase angle of the coupons in the fracturing water sample shifted toward the high-frequency region, while the phase angle of the coupons in the flowback water sample moved toward the low-frequency region. The test coupons in the fracturing water sample exhibited Vorberg impedance, which is induced by the concentration polarization and is associated with a reduced liquid-phase mass transfer rate (Chunan, 2008).

**Table 1:** Fitting results of EIS data for steel coupons immersed in a fracturing water system for 5 days.

Time (d)	*R*_s_ (Ω cm^2^)	*Q*_f_ (Ω^−1^ cm^−2^ s^n^)	*n*	*R*_f_ (KΩ cm^2^)	*Q*_dl_ × 10^−4^ (Ω^−1^ cm^−2^ s^n^)	*n*	*R*_ct_ (KΩ cm^2^)	*W* (Ω^−1^)
1	49.60 ± 1.32	4.84 ± 5.24	0.64 ± 0.37	0.05 ± 0.04	8.22 ± 5.26	0.60 ± 0.28	1.68 ± 0.68	4.33 ± 2.02
3	15.90 ± 1.53	1.70 ± 0.54	0.94 ± 0.06	0.14 ± 0.03	333.74 ± 577	0.45 ± 0.39	0.29 ± 0.10	20.6 ± 0.06
5	7.80 ± 0.10	1.47 ± 0.67	0.96 ± 0.06	0.17 ± 0.11	5.37 ± 4.01	0.85 ± 0.19	0.28 ± 0.15	13.6 ± 0.28

**Table 2:** Fitting results of EIS data for test coupons immersed in a flowback liquid system for 5 days.

Time (d)	*R*_s_ (Ω cm^2^)	*Q*_f_ (Ω^−1^ cm^−2^ s^n^)	*n*	*R*_f_ (KΩ cm^2^)	*Q*_dl_ × 10^−4^ (Ω^−1^ cm^−2^ s^n^)	*n*	*R*_ct_ (KΩ cm^2^)
1	11.64 ± 2.87	1.67 ± 1.00	0.93	0.68 ± 0.05	7.01 ± 1.44	0.41 ± 0.01	37.51 ± 10.09
3	8.91 ± 0.28	3.32 ± 4.04	0.91 ± 0.15	0.08 ± 0.12	5.26 ± 4.14	0.81 ± 0.16	6.22 ± 0.38
5	43.48 ± 2.91	55.1 ± 1.36	1.00	0.27 ± 0.01	57.4 ± 0.99	1.00	2.24 ± 0.01

### 3.4 Weight loss experiments

**Figure 7:**
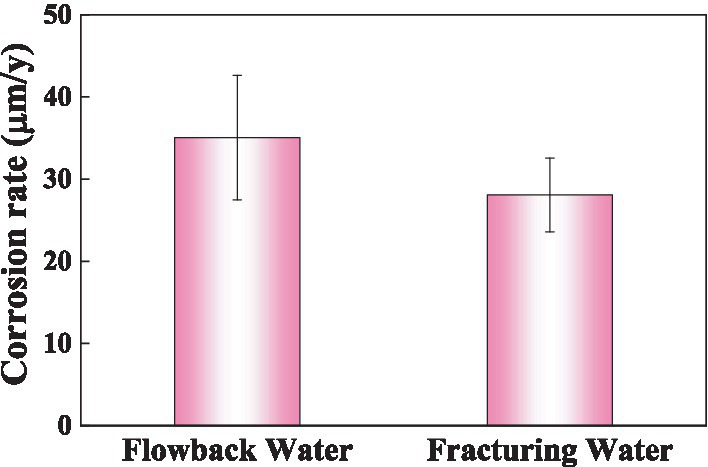
The corrosion rate of steel coupons after immersed in different media for 5 days.

Figure 7 shows the corrosion rate of the Q355 steel coupons in culture media inoculated with samples of fracturing water and flowback water as bacterial sources over a period of 5 days. All conditions, except for the bacterial source, remain consistent across both systems. The corrosion rates of steel coupons immersed in fracturing and flowback water inoculated culture media were 35.04 ± 7.57 mpy and 28.07 ± 4.49 mpy, respectively. The corrosion rate of the coupons in the flowback water was higher than in the fracturing water, exhibiting an increase of approximately 24.84%. This finding suggests that the corrosive microorganisms present in the fracturing water exhibit a greater corrosive effect on the Q355 coupons. The weight loss results indicated that the corrosion weight caused by the fracturing water may accounts for 75.16% during in 5 days’ immersion.

## 4 Discussion

### 4.1 The underground microbe community and their potential interaction

The corrosion of underground pipelines is influenced by a multitude of factors, including microorganisms, high temperature, high pressure and gas atmosphere, et al. Generally, microorganisms form biofilms on the steel surfaces and then develop into complex microbial communities (Flemming et al., 2024). The biological activities of microorganisms within the biofilm, such as the production of corrosive acids and the reduction and oxidation of certain elements, can lead to severe MIC and local corrosion problems. The underground microbes have metabolic interactions and jointly act on the corrosion of pipelines.

The genus *Desulfovibrio* is a typical corrosive bacterial strain and is also one of the most extensively studied SRB. *Desulfovibrio* are chemo-organotrophic organisms that utilize lactate, pyruvate, fumarate, succinate, malate as electron donors and carbon sources, and hydrogen as an electron donor. Heidelberg et al. (2004) studied the genome sequence and characteristics of the SRB strain *Desulfovibrio vulgaris* Hildenborough (NCIMB 8303) and found that hydrogenases and cytochromes may be involved in the hydrogen depolarization process of metals. The corrosive sulfides and organic acids, including like acetic acid, lactic acid, and pyruvic acid, produced by the metabolic processes of SRB indirectly lead to corrosion. The sites of maximum corrosion typically occur where intermittent oxidation reactions take place. *Desulfovibrio* spp. can use hydrogen, organic acids, and acetate as electron donors to reduce sulfate ions. Hydrogenases facilitates bacteria utilizing hydrogen, while the deletion of hydrogenase genes in *Desulfovibrio vulgaris* Hildenborough hinders the inorganic metabolic processes of SRB (Voordouw, 2002). Hydrogenases and cytochrome C facilitate the electron transport in many microbes. In the electron transport chain, cytochrome *c_3_* plays an important role in the intracellular electron transport chain, initially receiving electrons from the electron donor and passing them to other electron acceptors. Cytochrome *c_3_* is specifically responsible for providing electrons to the NiFe-hydrogenase.

It was reported that *Desulfovibrio* can symbiotically coexist with other microbes (Plugge et al., 2011; Sherar et al., 2011), facilitating direct electron transfer through nanowires. *Desulfovibrio vulgaris* strains containing hydrogenases can produce more sulfide ions when both lactate and iron blocks are present compared to when only lactate is present, with the excess sulfide ions being reduced from sulfate ions through cathodic hydrogen depolarization (Cordruwisch and Widdel, 1986). Duan et al. (2008) found that *Desulfovibrio* caledoniensis can obtain electrons from steel, accelerating the corrosion of steel (Lin, 2011; Yu et al., 2011). Additionally, *Desulfovibrio* can obtain electrons from the metal surface via the redox reduction of hydrogen and get electrons directly from metals or polarized metals via hydrogen redox reduction.

*Desulfomicrobium* was first discovered in the drain water and drilling mud of oil fields as early as 1999 (Leu et al., 1999), and it is believed to play an important role in the formation of sulfides. Dengler et al. (2023) isolated a novel interdomain consortia composed of methanogenic archaea and *Desulfomicrobium baculatum* from a mix biofilm in an oil well at the Corcovado National Park in Costa Rica. Both organisms can grow in pure cultures or stable co-cultures. Interspecies association can be achieved through the utilization of hydrogen, lactate, formate, and pyruvate as electron donors, with sulfate, thiosulfate, and sulfite serving as electron acceptors. Furthermore, Dias et al. (2008) isolated *Desulfomicrobium* salsuginis and *Desulfomicrobium* aestuarii from estuary and found that they possess specific potential for mercury methylation. *Desulfomicrobium macestii* can chemolithoautotrophically grow on H_2_ and CO_2_ through sulfate reduction. Actually, almost all species can grow by fermenting pyruvate, fumarate or malate. Almost all species possess hydrogenases, b-cytochromes and c-type cytochromes. *Desulfomicrobium* sp. exhibits thermophilic and heavy metal tolerance characteristics.

Additionally, *Acetobacterium* and *Desulfomicrobium* can utilize hydrogen (Honghong and Guoheng, 2017), *Acetobacterium psammolithicum* sp. nov. and *Desulfomicrobium hypogeium* sp. nov. have a competitive relationship in utilizing hydrogen in the subsurface rock layers, the competition among microorganisms that perform terminal electron acceptance processes in an anaerobic ecosystem. When competition occurs between microorganisms using respiratory electron acceptors, the thermodynamically favorable processes usually dominate (Honghong and Guoheng, 2017). When microorganisms that utilize respiratory electron acceptors compete with each other, thermodynamically favorable processes typically occur firstly than others. The thermodynamic favorability of a process is determined by the Gibbs free energy change associated with the redox reactions, which influences the microbial community structure and the progression of biogeochemical cycles. *Acetobacter* is a common bacterium in almost all anaerobic ecosystems, co-cultured with H_2_ and CO_2_ for metabolism. Thermodynamically, SRB and methanogenic bacteria may have more competitive advantages in using hydrogen or formate salts than the process of producing acetic acid (Plugge et al., 2011; Saha et al., 2020). In the underground gas well stations, there are special microenvironments and microorganisms with special functions in the subsurface sandstone. *Acetobacter* seems to have both autotrophic ability to produce acetate salts, and more importantly, to produce acetate salts from the degradation of low molecular weight organic compounds, which may provide carbon and electron donors for corrosion microorganisms (Honghong and Guoheng, 2017). Previous study (Zhang Y. et al., 2023) has found that the increase in seawater salinity leads to a decrease in *Acinetobacter* genus and an increase in the abundance of *Desulfomicrobium*. Besides, *Sphaerochaeta* was found in the Middle-viscosity fracturing water with an abundance of 1.21% and the flowback water-2 with 3.18%. *Sphaerochaeta* is known to consume the byproducts of petroleum biodegradation by other microorganisms, participate in biofilm formation, and supply H_2_ to methanogens and other members of the community (Bidzhieva et al., 2018). the cooperation of microbes might promote the gas and oil production.

The study revealed that bacterial communities in shale gas fields exhibit a high level of diversity. This diversity is manifested not only in the number of bacterial species but also in the wide range of their metabolic types. These bacteria include, but are not limited to SRB, IOB, NRB and saprophytic bacteria. The environment of shale gas fields is characterized by high temperature, high pressure, high salinity, and oxygen depletion, which poses challenges to the survival of bacteria. Bacterial communities in shale gas fields are typically found to be highly adapted to these extreme conditions. There were diverse metabolic types, including anaerobic metabolism, aerobic metabolism, fermentative metabolism, and photosynthesis. These various metabolic pathways enable bacteria surviving in the underground severe environments within shale gas fields (Supplementary Figure S2). The metabolic activities of those bacteria can produce corrosive metabolic substances, which can accelerate the corrosion of metal structures. Besides, some bacteria with the capability of degrading complex organic matter, which plays a positive role in enhancing gas recovery rates and reducing environmental pollution.

### 4.2 The trace of the corrosive microbes in the underground pipelines

**Figure 8:**
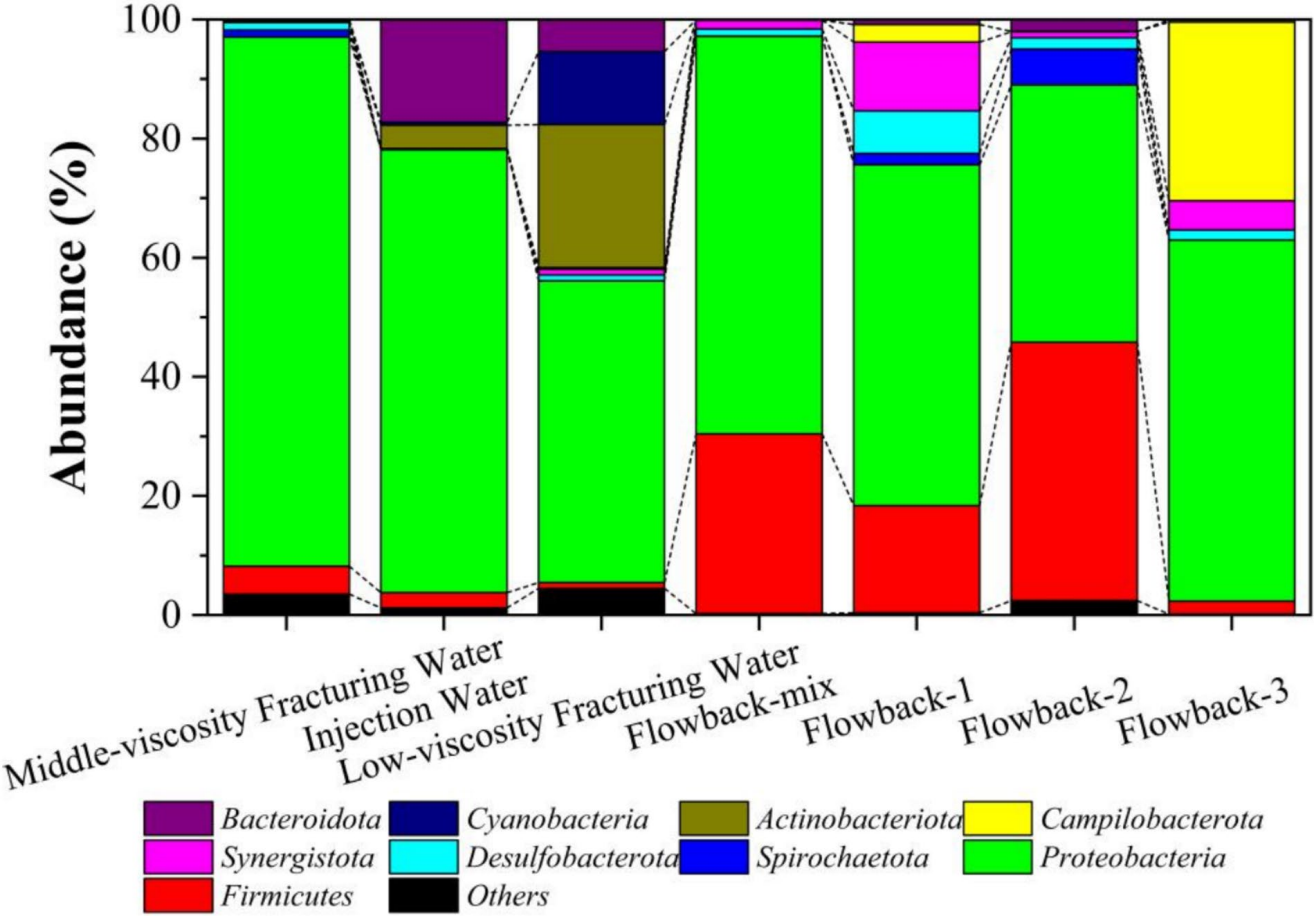
The abundance of microbes of the fracturing water and flowback water on the phylum level.

In the level of Phylum (Figure 8), *Proteobacteria* was the dominant phylum that must abundant bacteria in all solutions. The abundance of *Proteobacteria* in the aqueous solution, middle-viscosity fracturing water, low-viscosity fracturing water was 74.37, 88.84, and 50.63%. While the abundances of *Proteobacteria* in flowback water of flowback-mix, flowback-1, flowback-2, and flowback-3 were 66.78, 57.22, 43.23, and 60.65%, respectively. In injection water, the abundance of *Actinobacteriota* and *Cyanobacteria* was 3.90 and 0.53%, respectively. However, their abundance increased to 24.01 and 12.29% in low-viscosity fracturing water. It indicated that among the other components that make up the low viscosity fracturing water, *Actinobacteriota* and *Cyanobacteria* were found in high abundance. Nature river water was the main component of the injection water, this may be the main reason that *Cyanobacteria* was found in injection water. While in the flowback water, there was almost no existence (less than 0.01%) of *Cyanobacteria*. Besides, the abundance of *Bacteroidota* in injection water was 17.22%, while it decreased to 0.12 and 5.36% in middle-viscosity fracturing water and low-viscosity fracturing water, respectively. Duan (Yao et al., 2023) found that the *Proteobacteria*, *Cyanobacteria* and *Bacteroidota* were the dominant bacterial phyla in the rust layer biofilm and seawater. It should be noted that higher microbial community diversity was found in the low-viscosity fracturing water than the aqueous solution and middle-viscosity fracturing water.

**Figure 9:**
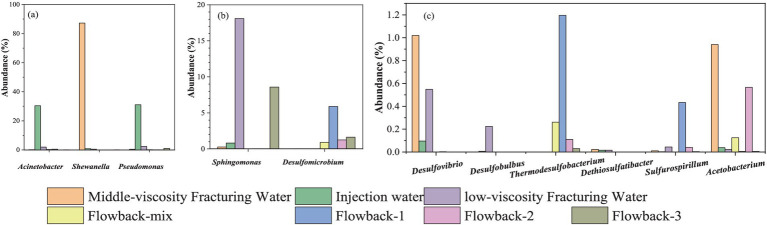
Abundance of several typical corrosive microbes in fracturing and flowback waters. **(a)** the abundance of *Acinetobacter*, *Shewanella* and *Pseudomonas*; **(b)** the abundance of *Sphingomonas* and *Desulfomicrobium*; **(c)** the abundance of *Desulfovibrio*, *Desulfobulbus*, *Thermodesulfobacterium*, *Dethiosulfatibacter*, *Sulfurospirillum* and *Acetobacterium*.

The abundance of several typical corrosion microorganisms were summarized in Figure 9 and their specific contents were listed in Supplementary Table S3. Typical SRB strains, such as *Desulfovibrio*, *Desulfomicrobium*, *Desulfobulbus*, and *Dethiosulfatibacter* were found in the fracking waters. In addition to the aforementioned SRB strains, *Desulfobacterota* and the thermophilic genus *Thermodesulfobacterium* were found in the flowback water. *Thermodesulfobacterium* species are chemo-organotrophic, utilizing hydrogen (H_2_) as an electron donor and carbon dioxide (CO_2_) as a carbon source for sulfate reduction, thereby facilitating chemolithoautotrophic growth. They are also capable of growth through fermentation of pyruvate and fumarate. As early as 1983, Hatchikian and Zeikus (1983) and Hatchikian et al. (1984) identified *Thermodesulfobacterium commune* from non-spore-forming thermophilic SRB and isolated a novel type of heterotrophic sulfide reductase enzyme, desulfofuscidin. Jeanthon et al. (2002) isolated *Thermodesulfobacterium*, a thermophilic, non-spore-forming, chemolithoautotrophic, sulfate-reducing bacterium from the hydrothermal vents of the Guaymas Basin, with a growth temperature range of 50–80°C and an optimal temperature of 75°C. *Thermodesulfobacteriaceae* (Hamilton-Brehm et al., 2013) were isolated from the Obsidian Pool in Yellowstone National Park, with a growth temperature range of 70–90°C and using hydrogen (H_2_) or formate as an electron donor and carbon dioxide (CO_2_) as a carbon source. It should be noted that *Thermodesulfobacterium* and *Desulfobacterota* are only found the in the flowback water but not in the fracturing water. The results indicated that the *Thermodesulfobacterium* and *Desulfobacterota* are indigenous microorganisms that may originate from the underground rock.

### 4.3 The corrosion caused by fracturing and flowback water, respectively

In the fracturing water, the underground microorganisms, such as *Pseudomonas*, *Acinetobacter*, *Shewanella* (Ikeda et al., 2021), and *Desulfovibrio*, participate in the corrosion process. Under anaerobic conditions, those microbes get electrons from steel directly or indirectly, accelerating the metal corrosion.

There are unique metabolic features in the flowback water (Figure 6). They may produce special metabolic products, such as surfactants, which can damage the protective film on the metal surface and intensify the corrosion.

In this present study, only a simple comparison of the steel corrosion rates was conducted via electrochemistry and weight loss experiments under normal pressure and room temperature conditions within a short period of 5 days.

However, it has to admit that the temperature and pressure of flowback water changed when it got out from underground. In addition to MIC, the underground pipeline also suffers corrosion problems such as erosion corrosion, stress corrosion, and carbon dioxide corrosion.

In addition, the redox potential of electrochemical reactions changes under high temperature and high pressure, according to thermodynamics, shown as follows (Cao and Zhang, 2002):


$$E=E^{{}^{\circ}}-\frac{0.0592}{n}\log\frac{{\left[C\right]}^{c}{\left[D\right]}^{d}}{{\left[A\right]}^{a}{\left[B\right]}^{b}}$$

Where, *E* and *E°* represents the redox potential at defined and standard conditions; n represents number of electrons transferred in the electrochemistry reaction; [A], [B], [C], and [D] represents the activity of redox mediators. The activity of the redox chemicals were influenced and controlled by environmental factors, such as pressure, temperature, concentrations, pH and others.

Taking the aforementioned factors into account, the actual corrosion of pipeline steels is likely to be even more severe. In the further work, long term verification and the investigation on the corrosion induced by the multiple microorganisms are needed for evaluating the corrosion impacts of fracturing water and flowback water.

## 5 Conclusion

The study of shale gas reservoir bacterial communities is an interdisciplinary field that involves geology, microbiology, biochemistry, and environmental science, among others. A deep understanding of the characteristics of these bacterial communities is important for effective management of gas reservoirs and environmental protection. MIC is an inevitable and significant factor in shale gas field exploitation and is one of the main cause of pipeline corrosion in these fields. In this study, microbial community analysis of fracturing water and flowback water was conducted to trace the source of corrosive microorganisms in underground pipeline. The results showed that corrosive SRB strains *Thermodesulfobacterium* and *Desulfobacterota* originated from the underground rocks. While other corrosive microorganisms such as *Desulfobulbus*, *Desulfomicrobium, Acinetobacter*, *Sphingomonas* and *Acetobacterium* may be introduced via the fracturing water. The weight loss of steel coupons in the inoculated media of fracturing water and flowback water showed distinct values. Under laboratory conditions of 5 days’ immersion period, the fracturing water contributed a significant proportion around 75.16% to the whole corrosion weight of the steel coupons. It is of great significance to understand the characteristics of these bacterial communities and find out the sources of corrosive microorganisms, so as to better control the source of corrosion and reduce the corrosion of shale gas fields.

## Data Availability

The datasets presented in this article are not readily available due to confidentiality concerns related to the Sichuan shale gas resource. Requests for accessing the datasets should be directed to the corresponding author.
