# Supplementary material for: Unraveling the source of corrosive microorganisms from fracturing water to flowback water in shale gas field: evidence from gene sequencing and corrosion tests
Source: Front Microbiol. 2025 Jun 18;16:1552006. doi: 10.3389/fmicb.2025.1552006 (PMC12213509; doi:10.3389/fmicb.2025.1552006)
Supplement: Supplementary file 1 [file Supplementary_file_1.doc]

**Electronic Supplementary Material**

**Unraveling the source of corrosive microorganisms from** **fracturing fluid to produced water in shale gas field: Evidence from gene expression and corrosion tests**

Yanran Wang^1^, Shaomu Wen^2^, Shibo Zhang^3^, Yongfan Tang^1^, Xi Yuan^1^, Fang Guan^4,5*^, Jizhou Duan^4,5^

(1.Research Institute of Natural Gas Technology, Petrochina Southwest Oil&Gasfield Company, Chengdu 610213, Sichuan, China; 2.Petrochina Southwest Oil&Gasfield Company, Chengdu 610051, Sichuan, China; 3.Sichuan Huayou Group Corporation, Limited, Petrochina Southwest Oil&Gasfield Company, Chengdu 610041, Sichuan, China; 4.^4^State Key Laboratory of Advanced Marine Materials, Chinese Academy of Sciences, Qingdao, 266071, China; 6.Institute of Marine Corrosion Protection, Guangxi Key Laboratory of Marine Environmental Science, Guangxi Academy of Sciences, 98 Daling Road, Nanning 530007, P. R. China)

* Correspondence:

Yanran Wang: 2858782013@qq.com;

Fang Guan: guanfang@qdio.ac.cn

Figure S1. Relative abundance of 16S rRNA gene sequences of the samples at the bacterial phylum level

Figure S2. KEGG pathway abundance of some selected metabolic pathways in flowback water

Table S1. The detailed information of the fracturing and flowback water samples

| Samples | Fracturing water | | | Flowback water | | | |
| --- | --- | --- | --- | --- | --- | --- | --- |
| Labbled names | Injection water | Middle-viscosity Fracturing water | Low-viscosity Fracturing water | Flowback -Mix | Flowback -1 | Flowback -2 | Flowback -3 |

Table S2. Alpha Diversity estimators for bacteria from drainage samples of hydraulic fracturing water using 16S rRNA gene sequencing.

|  | Chao1 | Goods coverage | PD whole tree | Shannon | Simpson |
| --- | --- | --- | --- | --- | --- |
| Injection water | 988 | 1.00 | 28.48 | 4.15 | 0.05 |
| Middle-viscosity Fracturing fluid | 260 | 1.00 | 7.59 | 1.12 | 0.59 |
| Low-viscosity Fracturing Fluid | 1344 | 1.00 | 42.20 | 4.58 | 0.05 |

Table S3. Abundance of selected typical corrosive microorganisms of fracturing waters and flowback water

| Function | Genus | Fracturing water | | | Flowback water | | | |
| --- | --- | --- | --- | --- | --- | --- | --- | --- |
|  |  | Middle-viscosity Fracturing water | Injection water | low-viscosity Fracturing water | flowback-mix | flowback-1 | flowback-2 | flowback-3 |
| SRB | *Desulfovibrio* | 1.02% | 0.10% | 0.55% | 0.00% | 0.00% | 0.00% | 0.00% |
|  | *Desulfomicrobium* | 0.02% | 0.01% | 0.04% | 0.91% | 5.90% | 1.26% | 1.62% |
|  | *Desulfobulbus* | 0.00% | 0.01% | 0.22% | 0.00% | 0.00% | 0.00% | 0.00% |
|  | *Thermodesulfobacterium* | 0.00% | 0.00% | 0.00% | 0.26% | 1.20% | 0.11% | 0.03% |
|  | *Dethiosulfatibacter* | 0.02% | 0.02% | 0.02% | 0.00% | 0.00% | 0.00% | 0.00% |
| SOB | *Sulfurospirillum* | 0.01% | 0.00% | 0.04% | 0.00% | 0.43% | 0.04% | 0.00% |
| ARB | *Acinetobacter* | 0.12% | 30.37% | 1.94% | 0.22% | 0.48% | 0.00% | 0.09% |
|  | *Acetobacterium* | 0.94% | 0.04% | 0.02% | 0.13% | 0.01% | 0.57% | 0.01% |
| NRB | *Achromobacter* | 0.00% | 0.00% | 0.03% | 0.00% | 0.00% | 0.00% | 0.00% |
|  | *Bacillus* | 0.00% | 0.03% | 0.02% | 0.00% | 0.00% | 0.00% | 0.00% |
|  | *Corynebacterium* | 0.00% | 0.00% | 0.05% | 0.00% | 0.00% | 0.00% | 0.00% |
|  | *Sphingomonas* | 0.27% | 0.82% | 18.09% | 0.00% | 0.01% | 0.03% | 8.59% |
| IRB | *Shewanella* | 87.26% | 0.83% | 0.45% | 0.01% | 0.01% | 0.02% | 0.09% |
|  | *Pseudomonas* | 0.40% | 31.04% | 2.46% | 0.15% | 0.09% | 0.06% | 1.08% |
